# Supplementary material for: Causal Relationship between Aging and Anorexia Nervosa: A White-Matter-Microstructure-Mediated Mendelian Randomization Analysis
Source: Biomedicines. 2024 Aug 16;12(8):1874. doi: 10.3390/biomedicines12081874 (PMC11351342; doi:10.3390/biomedicines12081874)
Supplement: Supplementary file 1 [file biomedicines-12-01874-s001.zip › Supplementary Figure/Figure S4/Singe SNP plot of MR analysis for TL to ubm-b-2022.pdf]

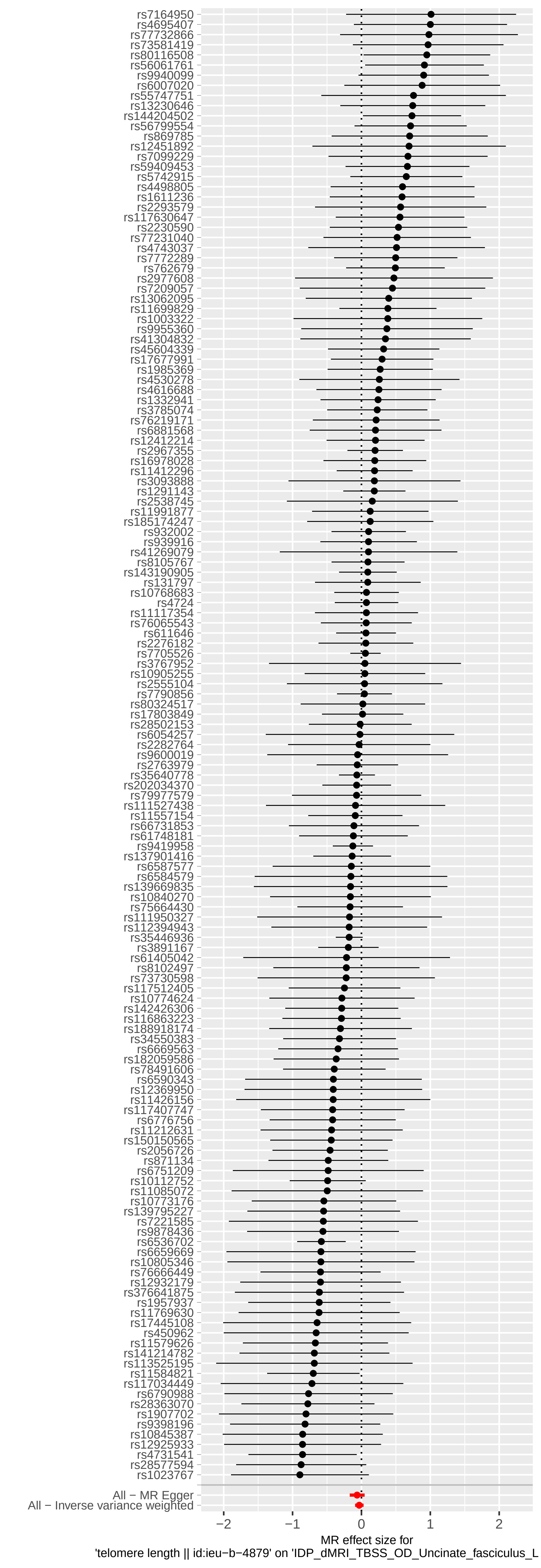
rs7164950
rs4695407
rs77732866
rs73581419
rs80116508
rs56061761
rs9940099
rs6007020
rs55747751
rs13230646
rs144204502
rs56799554
rs869785
rs12451892
rs7099229
rs59409453
rs5742915
rs4498805
rs1611236
rs2293579
rs117630647
rs2230590
rs77231040
rs4743037
rs7772289
rs762679
rs2977608
rs7209057
rs13062095
rs11699829
rs1003322
rs9955360
rs41304832
rs45604339
rs17677991
rs1985369
rs4530278
rs4616688
rs1332941
rs3785074
rs76219171
rs6881568
rs12412214
rs2967355
rs16978028
rs11412296
rs3093888
rs1291143
rs2538745
rs11991877
rs185174247
rs932002
rs939916
rs41269079
rs8105767
rs143190905
rs131797
rs10768683
rs4724
rs11117354
rs76065543
rs611646
rs2276182
rs7705526
rs3767952
rs10905255
rs2555104
rs7790856
rs80324517
rs17803849
rs28502153
rs6054257
rs2282764
rs9600019
rs2763979
rs35640778
rs202034370
rs79977579
rs111527438
rs11557154
rs66731853
rs61748181
rs9419958
rs137901416
rs6587577
rs6584579
rs139669835
rs10840270
rs75664430
rs111950327
rs112394943
rs35446936
rs3891167
rs61405042
rs8102497
rs73730598
rs117512405
rs10774624
rs142426306
rs116863223
rs188918174
rs34550383
rs6669563
rs182059586
rs78491606
rs6590343
rs12369950
rs11426156
rs117407747
rs6776756
rs11212631
rs150150565
rs2056726
rs871134
rs6751209
rs10112752
rs11085072
rs10773176
rs139795227
rs7221585
rs9878436
rs6536702
rs6659669
rs10805346
rs76666449
rs12932179
rs376641875
rs1957937
rs11769630
rs17445108
rs450962
rs11579626
rs141214782
rs113525195
rs11584821
rs117034449
rs6790988
rs28363070
rs1907702
rs9398196
rs10845387
rs12925933
rs4731541
rs28577594
rs1023767
All − MR Egger
All − Inverse variance weighted
−2
−1
0
1
2
MR effect size for
'telomere length || id:ieu−b−4879' on 'IDP_dMRI_TBSS_OD_Uncinate_fasciculus_L
